# Supplementary material for: A Replication Study Identified Seven SNPs Associated with Quantitative Traits of Type 2 Diabetes among Chinese Population in A Cross-Sectional Study
Source: Int J Environ Res Public Health. 2020 Apr 3;17(7):2439. doi: 10.3390/ijerph17072439 (PMC7177704; doi:10.3390/ijerph17072439)
Supplement: Supplementary file 1 [file ijerph-17-02439-s001.pdf]

International Journal of
Environmental Research and Public Health

MDPI

Supplemental Tables

Table S1. Hardy-Weinberg equilibrium test of T2D-associated loci.

Identified Association to	Loci	Chr	Reported Gene	Allele	Genotype	Genotype Freq	*P* Value
T2D	rs340874	1	PROX1	T/C	TT/CT/CC	524/712/247	0.872
	rs243021	2	BCL11A	A/G	AA/GA/GG	708/642/156	0.552
	rs2943641	2	IRS1	C/T	CC/CT/TT	1304/210/11	0.463
	rs3923113	2	GRB14	A/C	AA/CA/CC	1129/352/27	1.000
	rs7593730	2	RBMS1, ITGB6	C/T	CC/CT/TT	1058/430/35	0.304
	rs780094	2	GCKR	T/C	TT/TC/CC	365/742/355	0.601
	rs1470579	3	IGF2BP2	A/C	AA/CA/CC	860/566/91	0.945
	rs16861329	3	ST6GAL1	C/T	CC/TC/TT	896/530/78	1.000
	rs4607103	3	ADAMTS9	C/T	CC/CT/TT	606/679/227	0.111
	rs4858889	3	SCAP	A/G	AA/GA/GG	1091/363/28	0.830
	rs7612463	3	UBE2E2	C/A	CC/CA/AA	981/462/66	0.222
	rs831571	3	PSMD6	C/T	CC/CT/TT	605/716/196	0.507
	rs6815464	4	MAEA	C/G	CC/CG/GG	508/760/236	0.088
	rs459193	5	ANKRD55	G/A	GG/GA/AA	400/757/349	0.837
	rs10946398	6	CDKAL1	A/C	AA/CA/CC	503/785/219	0.002
	rs1535500	6	KCNK16	G/T	GG/GT/TT	424/755/329	0.877
	rs9470794	6	ZFAND3	T/C	TT/CT/CC	707/648/160	0.516
	rs2191349	7	DGKB, TMEM195	T/G	TT/GT/GG	632/631/211	0.009
	rs4607517	7	GCK	G/A	GG/GA/AA	918/501/76	0.495
	rs864745	7	JAZF1	T/C	TT/CT/CC	863/572/64	0.011
	rs972283	7	KLF14	G/A	GG/GA/AA	794/605/107	0.603
	rs13266634	8	SLC30A8	C/T	CC/TC/TT	443/798/268	0.006
	rs516946	8	ANK1	C/T	CC/TC/TT	1165/345/13	0.022
	rs896854	8	TP53INP1	C/T	CC/CT/TT	699/649/163	0.517
	rs10811661	9	CDKN2A, CDKN2B	T/C	TT/TC/CC	396/774/268	0.001
	**rs13292136**	**9**	**CHCHD9**	**C/T**	**CC/TC/TT**	**1522/4/15**	**<0.001**
	rs17584499	9	PTPRD	C/T	CC/CT/TT	1226/282/14	0.779
	rs2796441	9	TLE1	A/G	AA/AG/GG	536/702/240	0.704
	rs7041847	9	GLIS3	A/G	GG/AG/AA	406/780/312	0.087
	rs10886471	10	GRK5	C/T	CC/TC/TT	867/508/66	0.492

	rs10906115	10	CDC123, CAMK1D	A/G	AA/AG/GG	594/741/182	0.035
	rs11257655	10	CDC123	T/C	TT/CT/CC	479/752/256	0.203
	**rs12571751**	**10**	**ZMIZ1**	**A/G**	**AA/GA/GG**	**1229/56/227**	**<0.001**
	**rs1802295**	**10**	**VPS26A**	**C/T**	**CC/TC/TT**	**932/384/237**	**<0.001**
	rs5015480	10	HHEX	C/T	TT/CT/CC	997/453/41	0.253
	rs10830963	11	MTNR1B	C/G	CC/CG/GG	486/775/245	0.030
	rs1552224	11	CENTD2	A/C	AA/CA/CC	1283/238/9	0.738
	rs2237892	**11**	**KCNQ1**	C/T	CC/TC/TT	652/737/142	0.001
	**rs231362**	**11**	**KCNQ1**	**G/A**	**GG/GA/AA**	**1453/17/25**	**<0.001**
	rs5215	11	KCNJ11	C/T	TT/CT/CC	540/756/222	0.108
	rs10842994	12	KLHDC5	C/T	CC/CT/TT	999/488/44	0.094
	**rs1531343**	**12**	**HMGA2**	**G/C**	**GG/GC/CC**	**1503/14/10**	**<0.001**
	rs7961581	12	TSPAN8, LGR5	T/C	TT/CT/CC	924/527/66	0.450
	**rs1359790**	**13**	**SPRY2**	**G/A**	**GG/GA/AA**	**1310/167/34**	**<0.001**
	rs11634397	15	ZFAND6	A/G	AA/AG/GG	1239/251/20	0.077
	rs2028299	15	AP3S2	A/C	AA/CA/CC	964/505/69	0.758
	rs7172432	15	C2CD4A, C2CD4B	A/G	AA/GA/GG	578/736/200	0.170
	rs7178572	15	HMG20A	A/G	AA/AG/GG	583/738/191	0.078
	rs7403531	15	RASGRP1	C/T	CC/TC/TT	598/618/167	0.721
	rs1558902	16	FTO	T/A	TT/AT/AA	1154/307/26	0.273
	rs7202877	16	BCAR1	T/G	TT/GT/GG	993/481/52	0.563
	rs8050136	16	FTO	C/A	CC/CA/AA	1189/312/23	0.621
	rs4430796	17	HNF1B	A/G	AA/AG/GG	748/615/119	0.658
	rs12454712	18	BCL2	T/C	TT/CT/CC	479/754/286	0.754
	rs12970134	18	MC4R	G/A	GG/GA/AA	1013/407/44	0.710
	rs8090011	18	LAMA1	G/C	GG/CG/CC	751/547/114	0.316
	rs10401969	19	CILP2	T/C	TT/CT/CC	1298/226/14	0.237
	**rs3786897**	**19**	**PEPD**	**A/G**	**AA/GA/GG**	**378/822/279**	**<0.001**
	rs3794991	19	GATAD2A	C/T	CC/TC/TT	1301/169/8	0.369
	**rs4812829**	**20**	**HNF4A**	**G/A**	**GG/GA/AA**	**487/582/446**	**<0.001**
	**rs6017317**	**20**	**HNF4A**	**T/G**	**TT/GT/GG**	**410/710/168**	**<0.001**
**Birth weight**	rs724577	4	LCORL	C/A	CC/CA/AA	385/757/267	0.002
	rs4432842	5	5q11.2	T/C	TT/CT/CC	425/779/310	0.179
	rs6931514	6	CDKAL1	G/A	GG/AG/AA	332/745/293	0.001
	**rs1801253**	**10**	**ADRB1**	**C/G**	**CC/CG/GG**	**494/788/201**	**<0.001**
	rs1042725	12	HMGA2	T/C	TT/CT/CC	971/493/63	0.937

**BMI**	rs2568958	1	1p31	A/G	AA/AG/GG	1254/228/13	0.409
	rs574367	1	SEC16B	G/T	GG/GT/TT	931/476/50	0.283
	rs7561317	2	TMEM18	G/A	GG/GA/AA	1188/266/9	0.180
	rs16892496	8	TRHR	A/C	AA/CA/CC	388/760/338	0.377
	rs7832552	8	TRHR	C/T	CC/TC/TT	396/746/361	0.796
	rs11030104	11	BDNF, BDNF-AS	A/G	AA/GA/GG	395/737/319	0.494
	rs6265	11	BDNF, BDNF-AS	C/T	CC/CT/TT	389/768/306	0.046
	**rs925946**	**11**	**BDNF-AS**	**G/T**	**GG/GT/TT**	**1367/114/10**	**<0.001**
	rs9939609	16	FTO	T/A	TT/AT/AA	1163/302/22	0.614

P values < 0.001 are shown in bold for deviation from Hardy-weinberg equilibrium.

Table S2. Overview of investigated genetic variants associated with insulin resistance.

Loci	Chr	Reported Gene	RA	Other Allele	β	Identified by Association to	Reference
rs780094	2	GCKR	C	T	0.0583	type 2 diabetes	Ingelsson E et al. [1]
rs7593730	2	RBMS1, ITGB6	C	T	0.1044	type 2 diabetes	Qi L et al. [2]
rs3923113	2	GRB14	A	C	0.0677	type 2 diabetes	Kooner JS et al. [3]
rs2943641	2	IRS1	C	T	0.174	type 2 diabetes	Ohshige T et al. [4]
rs831571	3	PSMD6	T	C	-0.0896	type 2 diabetes	Chen M et al. [5]
rs4607103	3	ADAMTS9	C	T	0.0862	type 2 diabetes	Zeggini E et al. [6]
rs16861329	3	ST6GAL1	T	C	-0.1508	type 2 diabetes	Qi L et al. [2]
rs459193	5	ANKRD55	G	A	0.077	type 2 diabetes	Harder MN et al. [7]
rs972283	7	KLF14	G	A	0.0392	type 2 diabetes	Voight BF et al. [8]
rs17584499	9	PTPRD	T	C	0.4511	type 2 diabetes	Chang YC et al. [9]
rs10886471	10	GRK5	C	T	0.1133	type 2 diabetes	Huaixing Li et al. [10]
rs2028299	15	AP3S2	C	A	0.0392	type 2 diabetes	Kazakova EV et al. [11]
rs12970134	18	MC4R	A	G	0.077	type 2 diabetes	Bradnová O et al. [12]
rs12454712	18	BCL2	T	C	0.04	type 2 diabetes	Walford GA et al. [13]
rs1558902	16	FTO	A	T	0.0993	type 2 diabetes	Zheng Y et al. [14]
rs9939609	16	FTO	A	T	0.33	BMI	Zheng Y et al. [14]
rs896854	8	TP53INP1	T	C	0.0488	type 2 diabetes	Jia Liu et al. [15]
rs7561317	2	TMEM18	G	A	1.8116	BMI	Sandholt CH et al. [16]
rs6265	11	BDNF, BDNF-AS	T	C	0.0343	BMI	Daily JW et al. [17]
rs7832552	8	TRHR	T	C	0.102	Low lean body mass	Lunardi CC et al. [18]
rs16892496	8	TRHR	C	A	0.8065	Low lean body mass	Lunardi CC et al. [18]
rs724577	4	LCORL	C	A	-0.042	birth weight	Horikoshi M et al. [19]

Table S3. Overview of investigated genetic variants associated with insulin release.

Loci	Chr	Reported Gene	RA	Other Allele	β	Identified by Association to	Reference
rs340874	1	PROX1	C	T	0.0677	type 2 diabetes	Philips R et al. [20]
rs4607517	7	GCK	A	G	0.077	type 2 diabetes	Ingelsson E et al. [1]
rs2191349	7	DGKB, TMEM195	T	G	0.0488	type 2 diabetes	Hong K et al. [21]
rs516946	8	ANK1	C	T	0.0862	type 2 diabetes	Harder MN et al. [7]
rs5015480	10	HHEX	C	T	0.1398	type 2 diabetes	Philips R et al. [20]
rs10830963	11	MTNR1B	G	C	0.0953	type 2 diabetes	Wang Y et al. [22]
rs7403531	15	RASGRP1	T	C	0.0953	type 2 diabetes	Huaixing Li et al. [10]
rs7202877	16	BCAR1	T	G	0.1133	type 2 diabetes	Harder MN et al. [7]
rs4430796	17	HNF1B	G	A	0.1222	type 2 diabetes	Machado-Silva W et al. [23]
rs7612463	3	UBE2E2	C	A	0.0953	type 2 diabetes	Xu K et al. [24]
rs1535500	6	KCNK16	T	G	0.0952	type 2 diabetes	Nicholas C et al. [25]
rs7041847	9	GLIS3	A	G	0.0872	type 2 diabetes	Muller YL et al. [26]
rs5215	11	KCNJ11	C	T	0.0677	type 2 diabetes	Gjesing AP et al. [27]
rs864745	7	JAZF1	T	C	0.0953	type 2 diabetes	Grarup N et al. [28]
rs7961581	12	TSPAN8, LGR5	C	T	0.0862	type 2 diabetes	Grarup N et al. [28]
rs7172432	15	C2CD4A, C2CD4B	A	G	0.1013	type 2 diabetes	Grarup N et al. [29]
rs1552224	11	CENTD2	A	C	0.1044	type 2 diabetes	Nielsen T et al. [30]
rs13266634	8	SLC30A8	T	C	-0.0997	type 2 diabetes	Steinthorsdottir V et al. [31]
rs10946398	6	CDKAL1	A	C	-0.1628	type 2 diabetes	Gjesing AP et al. [27]
rs8090011	18	LAMA1	G	C	0.0296	type 2 diabetes	Matsuba R et al. [32]
rs1470579	3	IGF2BP2	C	A	0.1133	type 2 diabetes	Rodriguez S et al. [33]
rs243021	2	BCL11A	A	G	0.0862	type 2 diabetes	Anna Jonsson et al. [34]
rs2568958	1	1p31	A	G	1.3271	BMI	Xi B et al. [35]
rs6931514	6	CDKAL1	G	A	-0.051	birth weight	Feng Lu et al. [36]
rs10811661	9	CDKN2A,CDKN2B	T	C	0.1655	type 2 diabetes	Zeggini E et al. [37]
rs8050136	16	FTO	A	C	0.1356	type 2 diabetes	MR Wing et al. [38]
rs11634397	15	ZFAND6	G	A	0.0488	type 2 diabetes	Zhao Q et al. [39]

References

1. Ingelsson, E.; Langenberg, C.; Hivert, M.F.; Prokopenko, I.; Lyssenko, V.; Dupuis, J.; Mägi, R.; Sharp, S.; Jackson, A.U.; Assimes, T.L.; et al. Detailed Physiologic Characterization Reveals Diverse Mechanisms for Novel Genetic Loci Regulating Glucose and Insulin Metabolism in Humans. *Diabetes* **2010**, *59*, 1266–1275.
2. Qi, L.; Cornelis, M.C.; Kraft, P.; Stanya, K.J.; Linda Kao, W.H.; Pankow, J.S.; Dupuis, J.; Florez, J.C.; Fox, C.S.; Paré, G.; et al. Genetic variants at 2q24 are associated with susceptibility to type 2 diabetes. *Hum. Mol. Genet.* **2010**, *19*, 2706–2715.
3. Kooner, J.S.; Saleheen, D.; Sim, X.; Sehmi, J.; Zhang, W.; Frossard, P.; Been, L.F.; Chia, K.S.; Dimas, A.S.; Hassanali, N.; et al. Genome-wide association study in individuals of South Asian ancestry identifies six new type 2 diabetes susceptibility loci. *Nat. Genet.* **2011**, *43*, 984–989.
4. Ohshige, T.; Iwata, M.; Omori, S.; Tanaka, Y.; Hirose, H.; Kaku, K.; Maegawa, H.; Watada, H.; Kashiwagi, A.; Kawamori, R.; et al. Association of new loci identified in European genome-wide association studies with susceptibility to type 2 diabetes in the Japanese. *PLoS ONE* **2011**, *6*, e26911.
5. Chen, M.; Hu, C.; Zhang, R.; Jiang, F.; Wang, J.; Peng, D.F; Tang, S.S.; Sun X.; Yan J.; Wang S.Y.; et al. A variant of PSMD6 is associated with the therapeutic efficacy of oral antidiabetic drugs in Chinese type 2 diabetes patients. *Sci. Rep.* **2015**, *5*, 10701.
6. Zeggini, E.; Scott, L.J.; Saxena, R.; Voight, B.F.; Marchini, J.L.; Hu, T.; de Bakker, P.I.; Abecasis, G.R.; Almgren, P.; Andersen, G.; et al. Meta-analysis of genome-wide association data and large-scale replication identifies additional susceptibility loci for type 2 diabetes. *Nat. Genet.* **2008**, *40*, 638–645.
7. Harder, M.N.; Ribel-Madsen, R.; Justesen, J.M.; Sparsø, T.; Andersson, E.A.; Grarup, N.; Jørgensen, T.; Linneberg, A.; Hansen, T.; Pedersen, O. Type 2 Diabetes Risk Alleles NearBCAR1 and inANK1Associate With Decreased β-Cell Function Whereas Risk Alleles NearANKRD55 and GRB14Associate With Decreased Insulin Sensitivity in the Danish Inter99 Cohort. *J. Clin. Endocrinol. Metab.* **2013**, *98*, E801–E806.
8. Voight, B.F.; Scott, L.J.; Steinthorsdottir, V.; Morris, A.P.; Dina, C.; Welch, R.P.; Zeggini, E.; Huth, C.; Aulchenko, Y.S.; Thorleifsson, G.; et al. Twelve type 2 diabetes susceptibility loci identified through large-scale association analysis. *Nat. Genet.* **2010**, *42*, 579.
9. Chang, Y.C.; Chiu, Y.F.; Liu, P.H.; Shih, K.C.; Lin, M.W.; Sheu, W.H.; Quertermous, T.; Curb, J.D.; Hsiung, C.A.; Lee, W.J.; et al. Replication of genome-wide association signals of type 2 diabetes in Han Chinese in a prospective cohort. *Clin. Endocrinol.* **2012**, *76*, 365–372.
10. Li, H.X.; Gan, W.; Lu, L.; Dong, X.; Han, X.Y.; Hu, C.; Yang, Z.; Sun, L.; Bao, W.; Li, P.T.; et al. A Genome-Wide Association Study Identifies GRK5 and RASGRP1 as Type 2 Diabetes Loci in Chinese Hans. *Diabetes* **2013**, *62*, 291–298.
11. Kazakova, E.V.; Zghuang, T.W.; Li, T.T.; Fang, Q.X.; Han, J.; Qiao, H. The Gas6 gene rs8191974 and Ap3s2 gene rs2028299 are associated with type 2 diabetes in the northern Chinese Han population. *Acta Biochim. Pol.* **2017**, *64*, 227–231.
12. Bradnová, O.; Vejražková, D.; Vaňková, M.; Lukášová, P.; Včelák, J.; Stanická, S.; Dvořáková, K.; Bendlová, B. Metabolic and Hormonal Consequencies of the "Obesity Risk" MC4R Variant (rs12970134) in Czech Women. *Physiol. Res.* **2015**, *64*, S187–S195.
13. Walford, G.A.; Gustafsson, S.; Rybin, D.; Stančáková, A.; Chen, H.; Liu, C.T.; Hong, J.; Jensen, R.A.; Rice, K.; Morris, A.P.; et al. Genome-Wide Association Study of the Modified Stumvoll Insulin Sensitivity Index Identifies BCL2 and FAM19A2 as Novel Insulin Sensitivity Loci. *Diabetes* **2016**, *65*, 3200–3211.
14. Zheng, Y.; Huang, T.; Zhang, X.M.; Rood, J.; Bray, G.A.; Sacks, F.M.; Qi, L. Dietary Fat Modifies the Effects of FTO Genotype on Changes in Insulin Sensitivity. *J. Nutr.* **2015**, *145*, 977–982.
15. Liu, J.; Wang, L.; Qian, Y.; Dai, J.C.; Shen, C.; Jin, G.F.; Hu, Z.B. ; Shen, H.B. Association of 48 type2 diabetes susceptibility loci with fasting plasma glucose. *Diabetes Res. Clin. Pract.* **2018**, *139*, 114–121.
16. Sandholt, C.H.; Vestmar, M.A.; Bille, D.S.; Borglykke, A.; Almind, K.; Hansen, L.; Sandbæk, A.; Lauritzen, T.; Witte, D.; Jørgensen, T.; et al. Studies of metabolic phenotypic correlates of 15 obesity associated gene variants. *PLoS ONE* **2011**, *6*, e23531.
17. Daily, J.W.; Park, S. Interaction of BDNF rs6265 variants and energy and protein intake in the risk for glucose intolerance and type 2 diabetes in middle-aged adults. *Nutrition* **2017**, *33*, 187–194.
18. Lunardi, C.C.; Lima, R.M.; Pereira, R.W.; Leite, T.K.; Siqueira, A.B.; Oliveira, R.J. Association between polymorphisms in the TRHR gene, fat-free mass, and muscle strength in older women. *Age* **2013**, *35*, 2477–2483.

19. Horikoshi, M.; Yaghootka, H.; Mook-Kanamori, D.O.; Sovio, U.; Taal, H.R.; Hennig, B.J.; Bradfield, J.P.; St Pourcain, B.; Evans, D.M.; Charoen, P.; et al. New loci associated with birth weight identify genetic links between intrauterine growth and adult height and metabolism. *Nat. Genet*. **2013**, *45*, 76–82.
20. Philips, R.; Langston, L.; Hwang, H.; Vandergriff, T.; Trynosky, T.; Berlingeri-Ramos, A.C. Primary cutaneous histiocytoid carcinoma with distant metastasis. *J. Cutan. Pathol.* **2017**, *44*, 376–380.
21. Hong, K.; Chung, M.; Cho, S.B. Meta-analysis of genome-wide association study of homeostasis model assessment β cell function and insulin resistance in an East Asian population and the European results. *Mol. Genet. Genomics* **2014**, *289*, 1247–1255.
22. Wang, Y.; Nie, M.; Li, W.; Ping, F.; Hu, Y.; Ma, L.; Gao, J.; Liu, J. Association of six single nucleotide polymorphisms with gestational diabetes mellitus in a Chinese population. *PLoS ONE* **2011**, *6*, e26953.
23. Machado-Silva, W.; Tonet-Furioso, A.C.; Gomes, L.; Córdova, C.; Moraes, C.F.; Nóbrega, O.T. The rs4430796 SNP of the HNF1β gene associates with type 2 diabetes in older adults. *Rev. Assoc. Med. Bras*. **2018**, *64*, 586–589.
24. Xu, K.; Jiang, L.; Zhang, M.; Zheng, X.; Gu, Y.; Wang, Z.; Cai, Y.; Dai, H.; Shi, Y.; Zheng, S.; et al. Type 2 Diabetes Risk Allele UBE2E2 Is Associated With Decreased Glucose-Stimulated Insulin Release in Elderly Chinese Han Individuals. *Medicine* **2016**, *95*, e3604.
25. Vierra, N.C.; Dadi, P.K.; Jeong, I.; Dickerson, M.; Powell, D.R.; Jacobson, D.A. Type 2 Diabetes—Associated K+ Channel TALK-1 Modulates b-Cell Electrical Excitability, Second-Phase Insulin Secretion, and Glucose Homeostasis. *Diabetes* **2015**, *64*, 3818–3828.
26. Muller, Y.L.; Piaggi, P.; Chen, P.; Wiessner, G.; Okani, C.; Kobes, S.; Knowler, W.C.; Bogardus, C.; Hanson, R.L.; Baier, L.J. Assessing variation across 8 established East Asian loci for type 2 diabetes mellitus in American Indians: Suggestive evidence for new sex-specific diabetes signals inGLIS3andZFAND3. *Diabetes Metab. Res. Rev.* **2017**, *33*, e2869.
27. Gjesing, A.P.; Hornbak, M.; Allin, K.H.; Ekstrøm, C.T.; Urhammer, S.A.; Eiberg, H.; Pedersen, O.; Hansen, T. High heritability and genetic correlation of intravenous glucose- and tolbutamide-induced insulin secretion among non-diabetic family members of type 2 diabetic patients. *Diabetologia* **2014**, *57*, 1173–1181.
28. Grarup, N.; Andersen, G.; Krarup, N.T.; Albrechtsen, A.; Schmitz, O.; Jørgensen, T.; Borch-Johnsen, K.; Hansen, T.; Pedersen, O. Association Testing of Novel Type 2 Diabetes Risk Alleles in the JAZF1, CDC123/CAMK1D, TSPAN8, THADA, ADAMTS9, and NOTCH2 Loci With Insulin Release, Insulin Sensitivity, and Obesity in a Population-Based Sample of 4,516 Glucose-Tolerant Middle-Aged Danes. *Diabetes* **2008**, *57*, 2534–2540.
29. Grarup, N.; Overvad, M.; Sparsø, T.; Witte, D.R.; Pisinger, C.; Jørgensen, T.; Yamauchi, T.; Hara, K.; Maeda, S.; Kadowaki, T.; et al. The diabetogenic VPS13C/C2CD4A/C2CD4B rs7172432 variant impairs glucose-stimulated insulin response in 5,722 non-diabetic Danish individuals. *Diabetologia* **2011**, *54*, 789–794.
30. Nielsen, T.; Sparsø, T.; Grarup, N.; Jørgensen, T.; Pisinger, C.; Witte, D.R.; Diabetes Genetics Replication and Meta-analysis (DIAGRAM) Consortium; Hansen, T.; Pedersen, O. Type 2 diabetes risk allele near CENTD2 is associated with decreased glucose-stimulated insulin release. *Diabetologia* **2011**, *54*, 1052–1056.
31. Steinthorsdottir, V.; Thorleifsson, G.; Reynisdottir, I.; Benediktsson, R.; Jonsdottir, T.; Walters, G.B.; Styrkarsdottir, U.; Gretarsdottir, S.; Emilsson, V.; Ghosh, S.; et al. A variant in CDKAL1 influences insulin response and risk of type 2 diabetes. *Nat. Genet.* **2007**, *39*, 770–775.
32. Matsuba, R.; Sakai, K.; Imamura, M.; Tanaka, Y.; Iwata, M.; Hirose, H.; Kaku, K.; Maegawa, H.; Watada, H.; Tobe, K.; et al. Replication Study in a Japanese Population to Evaluate the Association between 10 SNP Loci, Identified in European Genome-Wide Association Studies, and Type 2 Diabetes. *PLoS ONE* **2015**, *10*, e126363.
33. Rodriguez, S.; Eiriksdottir, G.; Gaun, T.R.; Harris, T.B.; Launer, L.J.; Gudnason, V.; Day, I.N. IGF2BP1, IGF2BP2 and IGF2BP3 genotype, haplotype and genetic model studies in metabolic syndrome traits and diabetes. *Growth Horm. IGF Res.* **2010**, *20*, 310–318.
34. Jonsson, A.; Ladenvall, C.; Ahluwalia, T.S.; Kravic, J.; Krus, U.; Taneera, J.; Isomaa, B.; Tuomi, T.; Renström, E.; Groop, L.; et al. Effects of Common Genetic Variants Associated With Type 2 Diabetes and Glycemic Traits on α- and β-Cell Function and Insulin Action in Humans. *Diabetes* **2013**, *62*, 2978–2983.
35. Xi, B.; Takeuchi, F.; Meirhaeghe, A.; Kato, N.; Chambers, J.C.; Morris, A.P.; Cho, Y.S.; Zhang, W.; Mohlke, K.L.; Kooner, J.S.; et al. Associations of genetic variants in/near body mass index-associated genes with type 2 diabetes: A systematic meta-analysis. *Clin. Endocrinol.* **2014**, *81*, 702–710.

36. Lu, F.; Qian, Y.; Li, H.; Dong, M.; Lin, Y.; Du, J.; Lin, Y.; Chen, J.; Shen, C.; Jin, G.; et al. Genetic variants on chromosome 6p21.1 and 6p22.3 are associated with type 2 diabetes risk: A case—Control study in Han Chinese. *J. Hum. Genet.* **2012**, *57*, 320–325.
37. Zeggini, E.; Weedon, M.N.; Lindgren, C.M.; Frayling, T.M.; Elliott, K.S.; Lango, H.; Timpson, N.J.; Perry, J.R.; Rayner, N.W.; Freathy, R.M.; et al. Replication of Genome-Wide Association Signals in UK Samples Reveals Risk Loci for Type 2 Diabetes. *Science* **2007**, *316*, 1336–1341.
38. Wing, M.R.; Ziegler, J.M.; Langefeld, C.D.; Roh, B.H.; Palmer, N.D.; Mayer-Davis, E.J.; Rewers, M.J.; Haffner, S.M.; Wagenknecht, L.E.; Bowden, D.W. Analysis of FTO gene variants with obesity and glucose homeostasis measures in the multiethnic Insulin Resistance Atherosclerosis Study cohort. *Int. J. Obes.* **2011**, *35*, 1173–1182.
39. Zhao, Q.; Xiao, J.; He, J.; Zhang, X.; Hong, J.; Kong, X.; Mills, K.T.; Weng, J.; Jia, W.; Yang, W. Cross-sectional and longitudinal replication analyses of genome-wide association loci of type 2 diabetes in Han Chinese. *PLoS ONE* **2014**, *9*, e91790.

© 2020 by the authors. Submitted for possible open access publication under the terms and conditions of the Creative Commons Attribution (CC BY) license (http://creativecommons.org/licenses/by/4.0/).
